# A novel biodegradable film based on κ‐carrageenan activated with olive leaves extract

**DOI:** 10.1002/fsn3.1554

**Published:** 2020-06-07

**Authors:** Thamiris R. Martiny, Bruna S. Pacheco, Claudio M. P. Pereira, Andrés Mansilla, Maria S. Astorga–España, Guilherme L. Dotto, Caroline C. Moraes, Gabriela S. Rosa

**Affiliations:** ^1^ Department Chemical Engineering Federal University of Pampa Malafaia Brazil; ^2^ Department of Chemical Engineering Federal University of Santa Maria Santa Maria Brazil; ^3^ Laboratory of Lipidomic and Bio–Organic Federal University of Pelotas Centro Brazil; ^4^ Laboratoy of Macroalgas Antárticas and Subantárticas Universidad de Magallanes Punta Arenas Chile; ^5^ Department of Science and Natural Resources Región de Magallanes y de la Antártica Chilena University of Magallanes Punta Arenas Chile; ^6^ Department of Food Engineering Federal University of Pampa Malafaia Brazil

**Keywords:** active packaging, antimicrobial packaging, biopolymer, food packaging, lamb meat

## Abstract

This research focused on the development of carrageenan based biodegradable films incorporated with olive leaves extract (OLE). OLE microbial inhibition and its total phenolics (TP) were evaluated. Carrageenan films were produced by casting technique and were characterized by thickness, water vapor permeability (WVP), color, mechanical properties, and infrared spectroscopy. In order to apply as lamb meat packaging, the antimicrobial effect of the films was investigated. Results showed that OLE inhibited *E. coli* growth and presented excellent TP (41.40 mg_GAE_/g). Thicknesses of the film with OLE (CAR‐OLE) were approximately 28% higher than film without OLE. Addition of OLE reduced the WVP by approximately 54%. CAR‐OLE was less resistant to breakage and more flexible showing darker color. FTIR showed interaction of carrageenan with OLE. Results revealed that CAR‐OLE promoted 167‐fold reduction in initial count of aerobic mesophiles indicating shelf‐life extension of lamb meat and promising use as antimicrobial food packaging.

## Graphical abstract

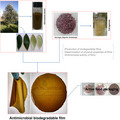

## 1 INTRODUCTION

Food packaging recent innovations beside ensuring product protection, safety and quality include meeting consumer demands for fresh, minimally processed, and preservative‐free foods and use of environmentally friendly materials. Currently, in order to meet the demand of packaging for the food industry, thousands of tons of synthetic polymers from petrochemical sources are produced, such as polyvinyl chloride (PVC). Despite their many advantages, most of these polymers are resistant to biodegradation, thus causing serious environmental problems with their disposal (Abdou & Sorour, 2014; Götz, Wani, Langowski, & Wunderlich, 2013). In this context, antimicrobial packaging based on biopolymers, which may be in the form of biodegradable films, is presented as an important and emerged alternative (Robertson, 2013). Antimicrobial packaging is a type of active packaging, which reduces, inhibits, or slows the growth of microorganisms, unlike conventional packaging, which there are basic functions, interacts with the same for its conservation, and extends shelf‐life while maintaining its quality. The biodegradable films become an antimicrobial package when antimicrobial agents are added in their formulation (Appendini & Hotchkiss, 2002; Labuza & Breene, 1989).

Carrageenans are biopolymers of the polysaccharide family, which have the ability to form good biodegradable films (Abdou & Sorour, 2014; Park, 1996). From the biotechnological point of view, sub‐Antarctic macroalgae are present in a variety and very large availability (Mansilla, Ávila, & Yokoya, 2012). In this context, several products of algal origin are explored. In particular, *Gigartina skottsbergii* is a red seaweed from Chile that is used in the extraction of carrageenan for food products (Mansilla et al., 2012). They belong to sulfated galactan hydrocolloids extracted from red algae (*Rhodophyta*) being generally distributed in three categories: kappa (κ)‐carrageenan, lambda (λ)‐carrageenan, and iota (ι)‐carrageenan (Williams, 2015). Carrageenans have properties that give it commercial application as additives used by the food industry, being gelling agents, emulsifiers, stabilizers, and thickeners (Williams & Phillips, 2009), but they have been gradually gaining recognition as a source of valuable materials, so it is necessary to intensify fundamental research, in order to discover novel functionality, taking full advantage of its potential, such as the production of biodegradable films.

The incorporation of bio compounds, such as phenolic compounds, in biodegradable films represents an interesting alternative. Aimed at the reduction of the use of chemical additives in the food industry, there has been a growing interest in the use of natural additives obtained from plant extracts from material considered as waste (Leuschner & Zamparini, 2002; Schmidt, Taylor, & Davidson, 2013). The olive leaf extract has been shown potential for application in biodegradable films, since olive leaves contain significant amounts of phenolics compounds, such as oleuropein, important factors for their antimicrobial capacity. (Benavente‐García, Castillo, Lorente, Ortuño, & Del Rio, 2000). Furthermore, another advantage of using olive leaves is that these leaves are considered as a by‐product of the olive oil industry, these can represent between 5% and 10% by weight, of the olive that enters for processing (Boudhrioua, Bahloul, Slimen, & Kechaou, 2008; El & Karakaya, 2009).

In the presented context, the development of biodegradable films from carrageenans and olive leaf extract can present a great potential in the diversification of the formulation of the films. Therefore, the aims of this study are develop and characterize biodegradable carrageenan films incorporated with olive leaves extract and evaluate their antimicrobial activity when used as lamb meat packaging.

## 2 MATERIALS AND METHODS

### 2.1 Materials

The carrageenan was extracted from the red algae *Gigartina skottsbergii* collected in Fuerte Bulnes, the sub‐Antarctic region of Magallanes, Chile (53º37'55.6"S, 70º55'17.9"W). Macroalgae specimens were collected manually during low tide at depths between 30 cm and 1.20 m from the intertidal and subtidal zone. The samples were transported in coolers filled with ice to laboratory refrigerators. In the laboratory, the samples were washed with Milli‐Q deionized water to remove epiphytes, salt, and foreign matter. The samples were dried at room temperature (20°C) for 5 days.

The extract of olive leaves was obtained from leaves collected from the type Arbequina (31º30'04.0"S, 53º30'42.0"W), located in Pinheiro Machado, Rio Grande do Sul, Brazil.

The chemicals used in this study were glycerol (Mistura da Terra, Brazil), used as plasticizer, and Folin Ciocalteu's phenol reagent (Sigma Aldrich). For microbiological, analyzes were used: nutrient broth, Müller–Hinton broth, brilliant green bile broth 2%, agar for counting plaque microorganisms—PCA agar and peptone (HiMedia), as well as distilled water, all sterilized in a vertical autoclave (Prismatec–CS). These analyses were conducted in a biological safety cabinet (Filterflux–Class II A2). All other reagents used were of analytical grade.

The bacterial strain *Escherichia coli* ATCC 11229 were supplied by Fiocruz–Oswaldo Cruz Foundation, Rio de Janeiro, Brazil. Stock cultures of the studied bacteria were grown on nutrient broth at 35°C for 24 hr before the tests.

### 2.2 Carrageenan extraction

The carrageenan extraction was performed according to (Pereira, Amado, Critchley, Velde, & Ribeiro‐Claro, 2009), (Sokolova et al., 2013), (Webber, Carvalho, Ogliari, Hayashi, & Barreto, 2012) and (Yermak et al., 2012). Red algae of the species *Gigartina skottsbergii*, previously dried were washed in tap water. 10 g of the sample was weighed and soaked in distilled water (800 ml) for 1 hr. The samples were then crushed with water in a blender. The solution was placed in a water bath at 74°C for 4 hr. Filtration for separation of the carrageenans from the residue was carried out in vacuum system. Nylon was used as filter medium. The filtered solution was oven dried at 60°C for 24 hr, thereby obtaining the solid carrageenan.

### 2.3 Preparation, characterization, and application of olive leaf extract (OLE)

The olive leaves were sanitized in running water, commercial solution of 2% to 2.5% sodium hypochlorite and sterilized distilled water. Subsequently, they were dried in an oven with forced air circulation at 40°C and comminuted in an analytical mill. The fraction that passed through in the 60 mesh sieve was used, obtaining a powder from the leaves. Thereafter, 10 g of the powder was macerated in 100 ml water and allowed to extract for 24 hr with shaking of 240 rpm and temperature of 25°C. The extract obtained was filtered by simple filtration with nylon (Nytex) with 100 µm of pore size and 44% open area.

For the determination of the total phenolic compounds, the spectrometric method adapted from (Singleton & Rossi, 1965) was used. An aliquot (0.5 ml) of OLE were mixed with 10 ml of distilled water and 1 ml of Folin Ciocalteu's. After 5 min, 8 ml of 7.5% (w/v) aqueous solution of sodium carbonate was added. The mixture was stored in the dark for two hours. Then the absorbance of the mixture was measured at wavelength of 765 nm with a spectrophotometer (Ultraspec1000, Amersham Pharmacia Biotech). The results of total phenolic compounds were expressed in milligrams of gallic acid equivalent per gram of dry matter. The analysis was performed in triplicate.

The bacterial inhibition assays were performed using the broth micro‐dilution method, using 96‐well microtitration plate, adapted to that described in the standard M07‐A10 of Clinical and Laboratory Standards Institute (CLSI) (Balouiri, Sadiki, & Ibnsouda, 2016; CLSI, 2015; Liu, McKeever, & Malik, 2017). Briefly, 135 μl of OLE were placed in a 96‐well plate along with 145 μl of sterile Muller–Hinton broth and 20 μl of the *E. coli* culture. The plate was incubated for a period of 16 hr at 35°C. Two absorbance readings at the wavelength of 630 nm (OD630) were recorded, one prior to incubation (0 hr) and one after the incubation period (16 hr), using a microplate reader (Celer–Polaris, Brazil). Wells without OLE and sterilized water were used as controls. Percent growth inhibition was calculated by Equation 1.

(1) $$I=\left[1-\left(\frac{{\text{OD}}_{\text{extract2}}-{\text{OD}}_{\text{extract1}}}{{\text{OD}}_{\text{control2}}-{\text{OD}}_{\text{control1}}}\right)\right].100$$

where *I* is the inhibition (%), ${\text{OD}}_{\text{extract2}}$ is the OD630 for the sample after the incubation period, ${\text{OD}}_{\text{extract1}}$ is the OD630 for the sample before the incubation period, ${\text{OD}}_{\text{control2}}$ is the OD630 for control after the incubation period, and ${\text{OD}}_{\text{control1}}$ is the OD630 for the control before the incubation period. Each experiment was repeated three times.

*E. coli* was chosen because other research has already demonstrated the antimicrobial action of olive leaf extract against this pathogen. Liu et al. (2017) investigated the antimicrobial effect of crude olive leaf extract and demonstrated that, at a concentration of 62.5 mg/ml, the extract almost completely inhibited *E. coli* growth. Markin, Duek, and Berdicevsky (2003) investigated the antimicrobial effect of olive leaves against bacteria, *E. coli* cells, subjected to a treatment with olive leaf extract showed complete destruction.

More than that, *E. coli* is recognized as an important causative agent of food‐borne disease. Especially for the scope of this work, meats are generally conducive to the development of bacteria. In particular, lamb meat due to its higher pH as compared to beef and pork, comprises an excellent substrate for the growth of pathogens including *Staphylococcus aureus, Listeria monocytogenes,* and *E. coli* (Karabagias, Badeka, & Kontominas, 2011).

### 2.4 Preparation and characterization of carrageenan biodegradable films

The carrageenan films were prepared according to the casting method, involving the formation of a film‐forming solution, which is subsequently dehydrated. Biodegradable films were prepared with carrageenan and plasticizer, as follows: 1% (w/v) of carrageenan, 37.5% (w/w) glycerol (based on the carrageenan mass), and 75% (v/v) of OLE (based on the final volume of the film solution in relation to the amount of water added). These proportions result in 0.5 g of carrageenan, 0.3 g of glycerol, 37.5 ml of extract, and 12.5 ml of water. The formula was dissolved under constant agitation (110 rpm) on hot plate and magnetic stirrer (Quimis‐Q261M23) at temperature of 70ºC for 15 min.

These conditions were determined in preliminary tests (Martiny, 2017), that were carried out to determine the most suitable plasticizer and OLE concentrations. The results demonstrated that films without the addition of plasticizer were brittle while those with high proportions of glycerol were sticky and difficult to remove from the plates. It was tested that films obtained with the OLE at concentrations below 75% (v/v) showed low or no antimicrobial activity. Carrageenan has this feature in small amounts to provide the desired effect, since it goes through the gelation process. In other studies, this proportion is usually employed. For example, Shojaee‐aliabadi, Hosseini, and Amin, (2013) used 1% carrageenan and 50% plasticizer. Glycerol was chosen as a plasticizer because it is one of the most widely used plasticizing agents in the production of biodegradable films, due to its compatibility and stability with the biopolymer chains. In addition, results in thinner films show very similar functional films attributes regarding their application as food wrappings and decrease film fragility (Fonseca et al., 2018; Saberi et al., 2016).

Finally, the film‐forming solution was poured into polystyrene Petri dishes (150 mm diameter) and biodegradable films were obtained by solvent evaporation in an oven with air circulation at 40ºC for 24 hr. After drying, film samples were peeled from Petri dishes and conditioned at room temperature for 48 hr in a desiccator containing sulfuric acid solution with a relative humidity of 50% before testing. The biodegradable carrageenan films produced with the olive leaf extract were designated [CAR‐OLE]. The biodegradable films of carrageenan without the addition of olive leaf extract were produced as controls [CAR‐control].

The biodegradable films thickness was measured by a digital micrometer (Insize‐IP65). Mean thickness was calculated from ten measurements taken at different locations on the biodegradable films samples.

Water vapor permeability (WVP) of biodegradable films was determined gravimetrically using the ASTM Standard E^96^/E96M (2012). Samples in the disks form were fixed on permeation cells containing granular anhydrous calcium chloride (CaCl_2_)–0% RH. These cells were placed in desiccators with 50% relative humidity at room temperature. Anhydrous calcium chloride mass gain was determined at 7 days during the experiment, and thus, it was possible to determine the water vapor transferred through the biodegradable film according to Equation 2.

(2) $$\text{WVP}=\frac{w_{\text{ab}}}{t}\frac{e}{A\Delta P}$$

where the *WVP* is the water vapor permeability (g/mPa^–1^s^–1^), and *w*_ab_ is the amount of absorbed moisture (g) which was measured by the difference of the initial weighing and final weighing of the experimental apparatus used in the analysis, *t* the time (s), *e* is the film thickness (m), *A* is the area of the exposed film surface (m^2^) and *ΔP* the partial pressure difference across the film (1,176,17 Pa at 294,31 K).

The tensile strength (TS) and elongation at break (EB) point of the films were determined using a Texturometer Analyzer (Stable Micro System–TA.XT plus) according to ASTM Standard D^882^–^09^ (2012). The samples were cut into 25‐mm‐wide and 100‐mm‐long strips and were clamped and deformed under tensile loading using a 50 N load cell with initial grip separation of 50 mm and cross‐head speed of 50 mm/min.

The color parameters of the biodegradable films were measured using the spectrophotometer (Konica Minolta–CM–2600D). The L*, a*, and b*standards were measured in 5 measurements at random points of the films. A white disk was used as standard. The color difference (ΔE) was calculated by Equation 3.

(3) $$\Delta E^{*}=\sqrt{{\left(L^{*}-L_{s}^{*}\right)}^{2}+{\left(a^{*}-a_{s}^{*}\right)}^{2}+{\left(b^{*}-b_{s}^{*}\right)}^{2}}$$

where $L^{*}$ , $a^{*}$ , and $b^{*}$ are the color parameter values of the standard and $L_{s}^{*}$ , $a_{s}^{*},$ and $b_{s}^{*}$ are the color parameter values of the sample. L* parameter ranges from 0 (black) to 100 (white). The a* parameter measures the degree of red (+a) or green (–a) color and the b* parameter measures the degree of yellow (+b) or blue (–b) color.

The Attenuated total reflectance Fourier transform infrared spectroscopy (FTIR–ATR) was used for the chemical characterization of carrageenan and to observe the structural interaction of carrageenan‐based films with added OLE. A Perkin‐Elmer spectrometer (UATR Two), in the range of 400 cm^–1^ to 4,000 cm^–1^, was used with 32 scans per spectrum and with a resolution of 4 cm^–1^. The samples were cut into small squares and then inserted into the sample portal of the FTIR–ATR apparatus to obtain the spectra to be analyzed.

### 2.5 Antimicrobial activity of biodegradable films in the packaging of lamb meat

The evaluation of the inhibitory effect of CAR–OLE films was performed by analyzing the growth of total coliforms and aerobic mesophiles during the storage of lamb meat. The sample of chilled lamb meat was purchased locally. The lamb meat was used fresh and raw, without any previous preparation. First, the microbiological analyzes (total coliforms and aerobic mesophiles) of the lamb meat sample at the initial time were performed. Three other samples were packaged in duplicate with different films—the first with the CAR‐control film, the second with the CAR–OLE film and the last with commercial film. The commercial film used was polyvinyl chloride (PVC), which was purchased from Royal Pack, Brazil. The lamb meat samples were packaged in a way that attempted to reproduce the conditions under which the fresh meat is marketed, that is, exposed in styrofoam trays and wrapped superficially with PVC film. This form of packaging allows the antimicrobial agents to be released gradually on the food surface by diffusion. The samples were kept packed for two days, and this period is generally given to chilled meat cuts.

Subsequently, the packed samples were stored at 7°C for 48 hr. After this 48 hr period, the microbiological (total coliforms and aerobic mesophilic) analyses of the lamb meat sample were performed. The most probable number (MPN) for enumeration of total coliforms and the standard plate count for analyzing total aerobic mesophiles (FAO, 1997) was used, following the guidelines of the American Public Health Association (APHA). Initially, a portion of each sample containing 25 g each were homogenized in a stomach type sample homogenizer (Marconi–MA440), with 225 ml of peptone water in sterile plastic bags, after serial dilutions. For the analysis of total coliforms, the results were based on the proportion of confirmed tubes for the three consecutive dilutions and expressed as MPN of total coliforms/g of sample. For the analysis of aerobic mesophiles, after incubation of the plates for 48 hr at 35°C, counts were made in plates, with microprocessor counter of colonies (EI, India), considering as results, the values obtained between 25 and 250 colonies. The results were expressed in colony forming units per gram of lamb meat (CFU/g).

### 2.6 Statistical analysis

Experimental data were analyzed by Statistica software (Stat Soft Inc., 10). The *Student* *t* test was applied for determining differences at 95% significance level.

## 3 RESULTS AND DISCUSSION

### 3.1 Phenolics and antimicrobial activity of olive leaves extract

The total phenolic content was 41.40 mg_GAE_/g (d.b.) greater than observed by Abaza et al. (2011) (16.52 mg_GAE_/g d.b) and within the superior end of the range reported by Ahmad‐Qasem et al. (2013) (21.6 to 43.4 mg_GAE_/g d.b.). In a recent new study, Rosa, Vanga, Gariepy, and Raghavan (2019) evaluate the effects of different extraction parameters on total phenolic compounds and antioxidant activity from olive leaves (*Olea europeae* L.), they demonstrated that olive leaves were a good source of phenolic compounds with high antioxidant activity.

The antimicrobial activity of olive leaves extract (OLE) was tested against an important food contaminant pathogenic bacterial, *E. coli*. At a concentration of 100 mg/ml, OLE completely inhibited the growth of *E. coli*, that is, the growth was inhibited by 100%. Certainly, the chemical composition of olive leaf extract conditioned the antimicrobial effect observed and the high concentration of phenolics compounds contributed for its antimicrobial properties. A similar result was reported by Liu et al. (2017). The authors investigated the antimicrobial of the crude extract of olive leaves and showed that at a concentration of 62.5 mg/ml, the extract inhibited in 95% the growth of *E. coli* and inhibited growth of *Listeria monocytogenes* and *Salmonella enteritidis* by 100%. However, Lee and Lee (2010) and Albertos et al. (2017) found no inhibitory activity of olive leaf extract against *E. coli*, even though there was inhibition against other bacterial species. These divergent results in relation to the inhibitory effect of the extract may be due to different methodologies of extraction and concentration of the extract, as well as the use of other solvents. In this work, the use of olive leaf extract, a by‐product of the production of olive oil and olives, was proposed as the source of antimicrobials for the production of active packaging.

### 3.2 Characteristics of biodegradable films

**FIGURE 1:**
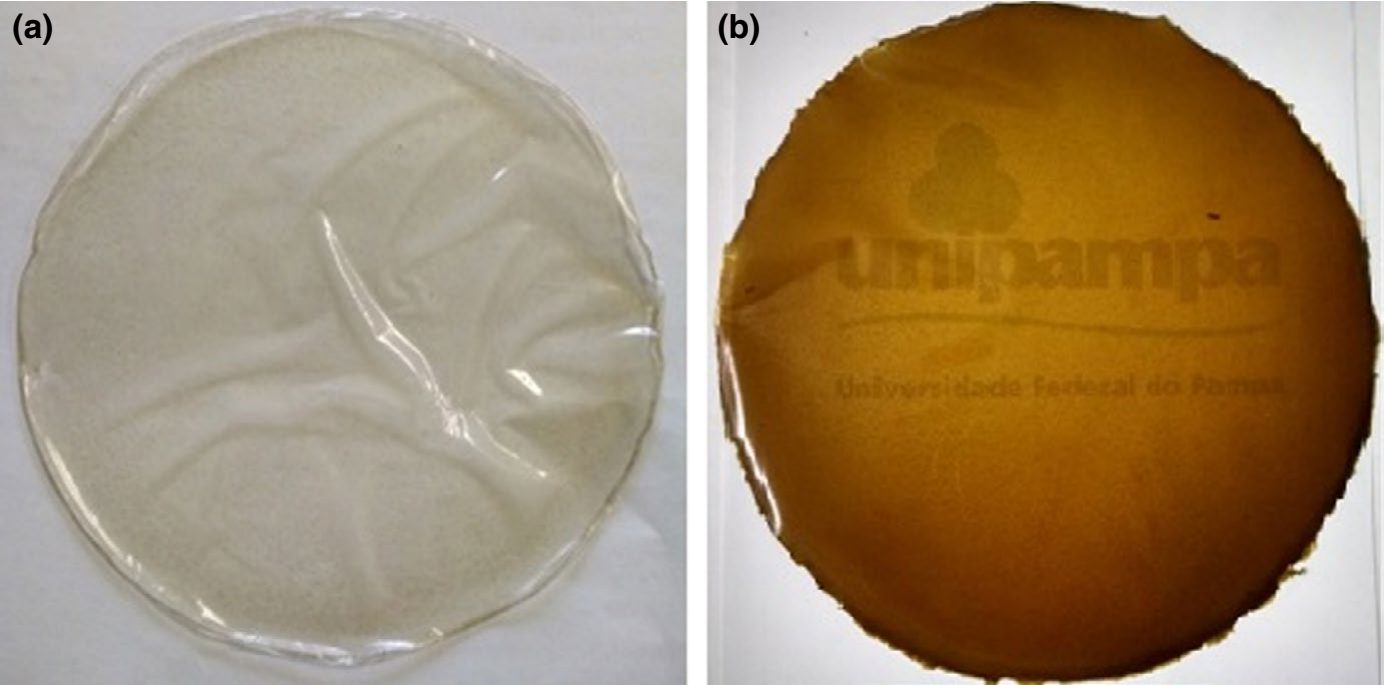
Biodegradable carrageenan films produced. (a) CAR‐control. (b) CAR‐OLE

The biodegradable films CAR‐control and CAR‐OLE appeared homogenous, uniform, nonbrittle, flexible and were easily removed from the support (plate) (Figure 1).

Table 1 shows the effect of OLE incorporation on carrageenan based films. The addition of the olive leaf extract in the biodegradable films of carrageenan caused an increase in the thickness of the films. This result was already expected due to the increase in mass of the filmogenic solution due to the incorporation of the extract (Sobral, 2000). These data were corroborated by Ma, Zhang, and Zhong (2016) who detected a significant increase in thickness after incorporation of antimicrobials into chitosan films.

**TABLE 1:** Thickness, WVP, and mechanical properties of biodegradable films and commercial PVC film

Film	Thickness (mm)	WVP (g/ms^–1^Pa^–1^)	Elongation at break (%)	Tensile strength (MPa)
CAR‐control	0.016 ± 0.002^a^	4.17 × 10^–11^ ± 2.38.10^–16a^	29.82 ± 5.13^a^	9.97 ± 0.67^a^
CAR‐OLE	0.058 ± 0.004^b^	2.26 × 10^–11^ ± 3.75 × 10^–12b^	54.15 ± 10.51^b^	7.76 ± 0.45^b^
PVC film	0.004 ± 0.001^c^	2.47 × 10^–13^ ± 3.67 × 10^–18c^	108.79 ± 15.71^c^	21.55 ± 3.22^c^

Note

Data reported are average values and ± mean deviation. Different letters represent significant differences (*p* < .05) between the mean obtained by the Student *t* test.

Abbreviations: CAR‐control, carrageenan biodegradable film; CAR‐OLE, Carrageenan biodegradable film with olive leaf extract.

The average thickness of carrageenan films showed significant difference (*p* > .05) regarding films without addition of extract and with addition of extract. The values were in the range of 0.016 and 0.058 mm. In a similar study, Silveira, Kranthi, Yvan, and Vijaya (2019) the results of thickness of biofilms were in the range of 0.097 to 0.162 mm and showed significant difference. These results are within those found by Martins et al. (2012a) (0.052 mm) who produced carrageenan films at the concentration of 1%, the same used in the present study, plasticized with glycerol. And very close to the result found by Rhim (2012) obtained 0.0582 mm thickness. The differences between present study and literature may be attributed to the fact that there are methodological differences in the preparation, composition, and proportions of the film‐forming solutions.

Table 1 shows the WVP values, which is a critical property for films intended for food packaging (Pereda, Amica, & Marcovich, 2012). It can be observed that the incorporation of OLE into biodegradable films caused a decrease in WVP. There was a statistically significant difference (*p* < .05) between CAR‐control WVP and CAR‐OLE. This is a positive result because low permeability values refer to higher food preservation. The water absorption capacity of the analyzed films depends, among other factors, on tortuosity. Tortuosity is used to describe diffusion in porous media and plays an important role in the water vapor transfer process (Pérez‐Gago & Krochta, 2001). The incorporation of OLE improved the barrier property of the carrageenan by increasing the path to be covered by the diffuser agent (water) as a function of the tortuosity imposed through the polymer matrix. Therefore, the permeant diffusivity was reduced resulting in a more tortuous path of the water vapor through the polymer matrix, resulting in a better water vapor barrier property of the film with the addition of OLE. Nazurah R. and Nur Hanani (2016) also found that the addition of plant essential oil at various concentrations in κ‐carrageenan based films significantly decreased WVP.

The results when compared to WVP of commercial PVC film have a higher WVP (Table 1). Several studies with carrageenan films have shown similar results, ranging from 5.8 × 10^–11^ to 2.18 × 10^–8^ g/ms^–1^Pa^–1^ (Karbowiak, Debeaufort, Champion, & Voilley, 2006; Martins et al., 2012; Paula et al., 2015; Rhim, 2012; Shojaee‐Aliabadi et al., 2014). In recent research, Albertos et al. (2017) reported that produced gelatin films with incorporated olive leaves extract showed WVP in the range of 1.83 × 10^–10^g/ms^–1^Pa^–1^–4.67 × 10^–10^g/ms^–1^Pa^–1^. These values are higher than found in this study, which may indicate that the use of the polymeric matrix of carrageenan is most advantageous.

It is observed from Table 1 that the addition of the extract caused significantly differences in mechanical properties of the films. Probably, this was due to the changes caused in the polymer structure with the incorporation of the extract, which conferred in additional plasticizer effect. (Pérez‐Gago & Rhim, 2013) confirmed this trend, and they stated that biodegradable films based on polysaccharides with the incorporation of lipids can improve the performance of the film, such as permeability, flexibility, and strength characteristics. The films presented moderate elongation and poor tensile strength according to the classification of Krochta and Mulder‐Johnston (1997). Martins et al. (2012a) obtained 16.18% elongation and 19.95 MPa for tensile strength for carrageenan films. Shojaee‐Aliabadi et al. (2014) produced carrageenan films (1%), the same proportion used in the present study, and plasticized with glycerol, with the result of elongation being 36.46% and rupture stress 26.29 MPa. Paula, Benevides, Cunha, Vit, et al. (2015) prepared carrageenan films and obtained as a result of elongation the value of 2.54% and tensile strength 11.64 MPa. The data reported in the literature for tensile strength are generally superior to those obtained for biodegradable films made with and without the addition of OLE. This occurrence may be due to differences in film thickness, conditions of storage, adaptations of the method, and mainly by the proportion of plasticizer used. It should be noted that normally a high tensile strength of the formulated film is required; however, the flexibility of the film, indicated by the elongation, is a very important parameter. That is, the longer the film elongation, the better its applicability as packaging will be (Teck Kim, Min, & Won Kim, 2013).

Table 2 shows the results for the parameters L*, a*, and b* for biodegradable films and for PVC film. The color of the film is an important factor for consumer acceptance. The films without OLE appeared clear, as did PVC films, both presented values of L* close to 100, which indicates the high transparency of the films. Already, the OLE films added showed a brownish‐green color.

**TABLE 2:** Optical properties of carrageenan films formulated with and without the addition of OLE and PVC film

Film	L*	a*	b*
CAR‐control	93.04 ± 0.26^a^	–0.07 ± 0.024^a^	5.59 ± 0.27^a^
CAR‐OLE	46.42 ± 0.53^b^	16.90 ± 0.26^b^	73.11 ± 0.51^b^
PVC	95.81 ± 0.78^c^	–0.14 ± 0,012^c^	1.088 ± 0.1464^c^

Note

Data reported are average values and ± mean deviation. Different letters represent significant differences (*p* < .05) between the mean obtained by the Student *t* test.

Abbreviations: CAR–control, carrageenan biodegradable film; CAR‐OLE, carrageenan biodegradable film with olive leaf extract.

As shown in Table 2, the addition of extract significantly decreases the L* parameter; that means, its transparency decreases. And the parameters a* and b* increased significantly with the addition of the extract, getting both positive, indicating the predominance of red and yellow component, respectively. The significant increase in b* can be attached to the phenolic compounds in olive leaf extract, which can absorb light of low wavelength. Similar behavior was observed by Shojaee‐Aliabadi et al. (2014) for embedded carrageenan films with essential oils, in which it was also reported for films carrageenan (1%) plasticized with glycerol, L*= 88.41, a*= –0.27 and b*= 0.86. Incorporated films with extract differed from the control by approximately 80%, as indicated by *ΔE.*

**FIGURE 2:**
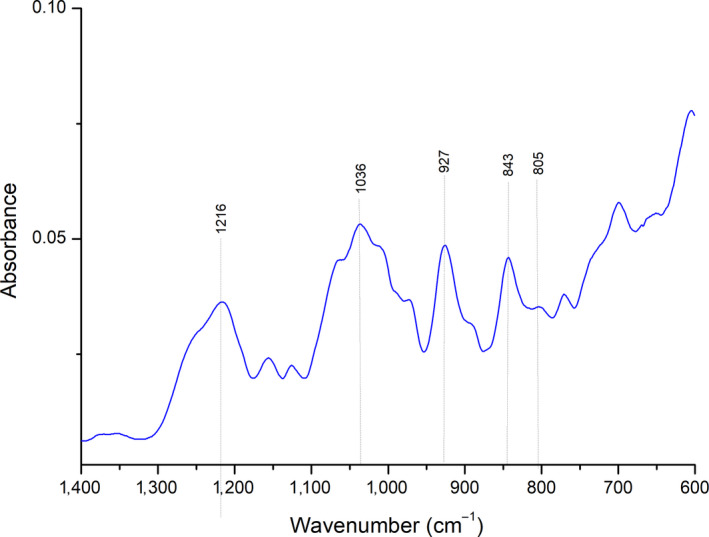
FTIR‐ATR extracted carrageenan

The FTIR–ATR spectrum of carrageenan extracted from the red algae of the species *Gigartina skottsbergii* is presented in Figure 2. This chemical characterization of carrageenan is important to development of applications based on unique intrinsic properties, in the case of this research, for the application in biodegradable films. Variations in the components of the carrageenan structure influence the hydration, strength, texture, and melting of the gel (dos Pereira, 2004).

The analyzed spectrum had strong absorption bands in the region of 1000–1100 cm^–1^, characteristic of polysaccharides, the region between 1,010 and 1,080 cm^–1^ is attributed to the glycosidic bonds present in carrageenans. The spectrum shows bands of strong absorption in the region 927 cm^–1^ (C–O of 3,6–anhydrogalactose) and in the region 843 cm^–1^ (C–O–SO_4_ of galactose‐4‐sulfate), typical of κ‐carrageenan. It also shows reduced absorbance in the 805 cm^–1^region, which is associated with the sulfate group of the 3,6‐anhydrogalactose unit, which means the presence of small amounts of ι‐carrageenan. It does not present a large band in the spectral region 820–830 cm^–1^and neither bands in the regions of 1,027 cm^–1^ and 867 cm^–1^, which are characteristics of λ‐carrageenan which indicates the presence of very small amounts or even absence of λ‐carrageenan (L. Pereira et al., 2009; L. Pereira, Sousa, Coelho, Amado, & Ribeiro‐claro, 2003). The obtained FTIR–ATR spectrum indicated that the carrageenan extracted from *Gigartina skottsbergii* is predominantly κ‐carrageenan withι‐carrageenan residues. The κ‐carrageenan corresponds to a less sulfated polymer and leads to a strong elastic gel and is less soluble than the other types of carrageenan because it has hydrophobic properties (dos Pereira, 2004). These results indicate the possibility of obtaining biodegradable films with excellent physical properties.

**FIGURE 3:**
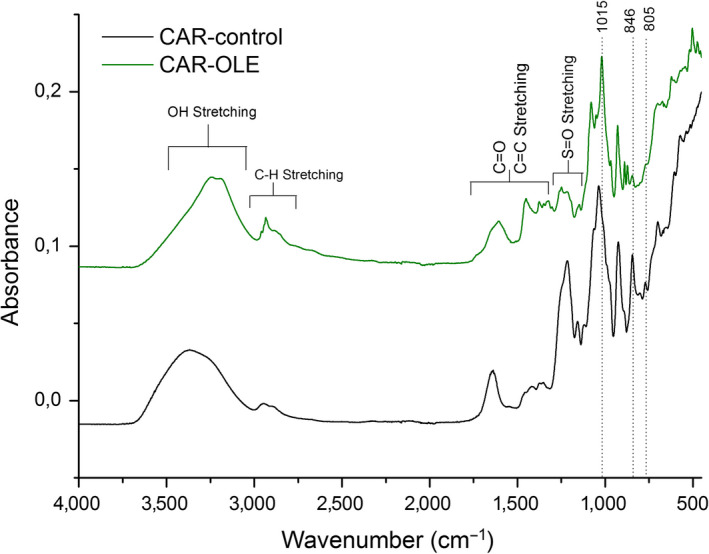
FTIR‐ATR spectra of carrageenan biodegradable films control (CAR‐control) and carrageenan biodegradable films with olive leaf extract (CAR‐OLE)

Figure 3 shows the FTIR–ATR spectra of carrageenan biodegradable films. With the analysis of the generated spectra, there was a displacement of the absorption bands of the CAR‐OLE film in relation to the CAR‐control film.

Both spectra show a broad band ranging from 3500–3100 cm^–1^, which is attributed to the O–H stretching vibration formed by the hydroxyl group of the polysaccharide and water. This range is more pronounced in the CAR‐OLE film because, in addition to the hydroxyl group of the polysaccharide and water, the O–H stretch relative to oleuropein and other compounds that are present in the extract. The proximal analysis of a variety of olive leaves performed by Boudhrioua et al. (2008) revealed that on average there are 40.49% carbohydrates. The broad band around 2,800 and 3,000 cm^–1^ was assigned to C–H stretching, it is also more pronounced in the CAR‐OLE film spectrum, due to additional groups extract. The region 1800–1500 cm^–1^ corresponding to stretching vibration of C = O and C = C (ester, acid, carboxylate, aromatic ring). In particular, for the CAR‐OLE the range 1500–1200 cm^–1^ is very complex, in which CH and OH deformation vibrations and CO stretching vibrations (phenols) can be found. Intense bands between 1,150 and 950 cm^–1^ correspond mainly to C–O stretching vibration endocyclic and exocyclic carbohydrate (Aouidi et al., 2012). The spectra of the films show several bands, dos Pereira (2004) indicates the range bands of the characteristic of each carrageenan type. It is important to highlight a band at 1215/1248 cm^–1^ sulfate ester groups corresponding to a band at 924/928 cm^–1^ assigned to the 3,6‐anhydrogalactose group, a band at 846 cm^–1^ corresponding galactose‐4‐sulfate and a band at 805 cm^–1^ corresponding to 3,6 anhydro D‐galactose‐2‐sulfate, κ‐carrageenan feature with small amounts of ι‐carrageenan. L. Pereira et al. (2003) also found intense bands at 845 cm^–1^ and 930 cm^–1^ characteristics of κ–carrageenans. The displacement of the CAR‐OLE film absorption bands relative to the CAR–control film may indicate that there was a miscibility between the carrageenan biopolymer matrix and the olive leaf extract and may possibly lead to changes in film properties such as barrier and mechanics.

## 4 ANTIMICROBIAL ACTIVITY OF BIODEGRADABLE FILMS IN THE PACKAGING OF LAMB MEAT

The lamb meat was checked for the microorganisms at the initial time, and the result was 100 MPN of total coliforms/g. After two days of storage, the results of 9.3–46 MPN of total coliforms/g of lamb meat for the sample packed with CAR‐OLE and > 110 MPN of total coliforms/g of lamb meat packed with CAR‐control. From the analysis of total coliforms, it was obtained that the lamb meat sample packed with CAR‐OLE had the microbial population decreased in relation to the initial population; the packaging showed inhibitory activity in the packed meat product. The sample packed with the CAR‐control did not stop the proliferation of the microorganisms, presenting a MPN of total coliforms/g of lamb meat higher than the one obtained initially. In this regard, Husna et al., (2019) also found promising results when packaging meat patties with carrageenan films incorporated with α‐tocopherol, lipid oxidation was delayed in meat patties wrapped with antioxidant films during storage. The results on the growth of aerobic mesophiles in the lamb meat at the initial time were 7.3 × 10^5^ ± 1.56 × 10^5^ CFU.g^–1^ and 7.10 × 10^4^ ± 3.39 × 10^1^ CFU.g^–1^ for samples packed with the biodegradable films of carrageenan and PVC, respectively. The final concentrations of aerobic mesophiles in sample after the two days of storage were 4.37 × 10^3^ ± 1.26 × 10^3^ CFU.g^–1^ for CAR‐OLE packaged samples, of 4.70 × 10^4^ ± 5.57 × 10^3^ CFU.g^–1^ for samples packed with CAR‐control and 1.36 × 10^3^ ± 1.94 × 10^2^ CFU.g^–1^ for samples packed with PVC film. The results show a significant difference in the aerobic mesophile count between the packaged samples with CAR‐OLE and CAR‐control, both packages reduced the initial count of micro‐organisms that were in the food–that is, the packaging with the carrageenan base had an inhibitory action. The addition of olive leaf extract in the formulation of the biodegradable carrageenan films significantly increased the inhibition, reducing the counting of aerobic mesophiles by 167 times, based on the initial count, while the biodegradable carrageenan film without addition of extract reduced only 15 times. The efficiency of CAR‐control can be attributed to its location on the surface of lamb meat where the main microbial concentrations occur. The PVC film does not have any additives that promote antimicrobial action, as does the CAR‐control film. In contrast, CAR‐OLE has the extract of olive leaves, which is rich in phenolic compounds, which are known to have antimicrobial action.

Several researchers have already shown that OLE can inhibit the growth of microorganisms in vitro assays. However, in this study, it has been demonstrated that biodegradable films with OLE can reduce the growth of aerobic mesophiles and total coliforms in foods, specifically, in lamb meat. The application of CAR‐OLE would provide an additional barrier to the growth of aerobic mesophiles and total coliforms. These results indicated that OLE, a by‐product of the olive oil industry, could be used in biodegradable films for application as active packaging.

Other researchers have also been successful in the preparation of active biodegradable packaging in food applications. Correa et al. (2017) prepared biodegradable Polyhydroxybutyrate/Polycaprolactone films and its nanocomposites activated with nisin showed potential for their application in processed meat packaging. Seol, Lim, Jang, Jo, and Lee (2009) evaluated the inhibition of carrageenan films supplemented with potassium sorbate plus EDTA when used in chicken breast packaging. They found that the films showed the effect of inhibition against total aerobic bacteria and *E. coli* during storage at 5°C; in addition, they claimed that further study should be done to investigate other applications for various meat products and different microorganisms due to the effectiveness found in the preparation of carrageenan‐based films.

More recently, Silveira et al. (2019) develop biodegradable carrageenan films with antioxidant properties by incorporating of olive leaf extract. The total phenolic compounds and antioxidant activity of films significantly increased with an increase in olive leaf extract concentration. The authors concluded that the incorporation of natural antioxidants appears to be a potential strategy to add additives into packaging material suitable for food products.

Contrary to some cited references that used a chemical preservative to confer the additional antimicrobial activity, or synthetic polymer in the formulation of the films, the present study used the extract of olive leaves (natural product), that in the same way promoted an inhibitory effect when applied in biodegradable films based on carrageenan. Thus, the extract of olive leaves becomes a promising in the formulation of biodegradable films.

## 5 CONCLUSIONS

The development of this research allowed the elaboration and characterization of active biodegradable films, as well as their application in the conditioning of lamb meat. The extract produced in this study from olive leaf constitutes a promising alternative for valorizing olive by‐products.

The results indicate that the addition of OLE in biodegradable films based on carrageenan promote changes in film properties and confer antimicrobial activity to them against aerobic mesophiles and total coliforms. OLE was highly effective against *E. coli*. The impact of the addition of OLE on the mechanical properties and WVP of the biodegradable carrageenan films was positive, as it increased the elongation at break, decreased the tensile strength and decreased the WVP. For the color analysis, the films produced with OLE presented a tendency to red and yellow coloration and differentiated from the control in approximately 80%. CAR–OLE reduced the growth of aerobic mesophiles in lamb meat during storage. The results of this study suggest that OLE as a natural antimicrobial has potential for use in biodegradable carrageenan films, which have been shown to be suitable for packaging according to the characterization results. The films developed in this study have a great prospect of application in the sector of active food packaging.
